# Real‐time assessment of swallowing sound using an electronic stethoscope and an artificial intelligence system

**DOI:** 10.1002/cre2.531

**Published:** 2022-01-11

**Authors:** Kazuma Suzuki, Yoshitaka Shimizu, Shinichiro Ohshimo, Kana Oue, Noboru Saeki, Takuma Sadamori, Yasuo Tsutsumi, Masahiro Irifune, Nobuaki Shime

**Affiliations:** ^1^ Department of General Dentistry Hiroshima University Hospital Hiroshima Japan; ^2^ Department of Dental Anesthesiology, Graduate School of Biomedical and Health Sciences Hiroshima University Hiroshima Japan; ^3^ Department of Emergency and Critical Care Medicine, Graduate School of Biomedical and Health Sciences Hiroshima University Hiroshima Japan; ^4^ Section of Dental Anesthesiology, Department of Oral & Maxillofacial Surgery and Oral Medicine Hiroshima University Hospital Hiroshima Japan; ^5^ Department of Anesthesiology and Critical Care, Graduate School of Biomedical and Health Sciences Hiroshima University Hiroshima Japan

**Keywords:** AI, cervical auscultation, deglutition, dysphagia, electronic stethoscope

## Abstract

**Objectives:**

Daily assessments of swallowing function and interventions such as rehabilitation and dietary adjustments are necessary to improve dysphagia. Cervical auscultation is convenient for health care providers for assessing swallowing ability. Although this method allows for swallowing sound evaluations, sensory evaluations with this method are difficult. Thus, we aimed to assess swallowing sound by the combined use of an electronic stethoscope and an artificial intelligence (AI) system that incorporates sound recognition.

**Material and Methods:**

Herein, 20 fifth‐year dentistry student volunteers were included; each participant was drank 10 ml and then 20 ml of water in different positions (sitting and supine). We developed an algorithm for indexing bolus inflow sounds using AI, which compared the swallowing sounds and created a new index.

**Results:**

The new index value used for swallowing sound was significantly higher in men than in women and in the sitting position than in the supine position. A software for acoustic analysis confirmed that the swallowing index was significantly higher in men than in women as well as in the sitting position than in the supine position. These results were similar to those obtained using the new index. However, the new index substantially differed between sexes in terms of posture compared with effective sound pressure.

**Conclusions:**

We developed a new algorithm for indexing swallowing sounds using a stethoscope and an AI system, which could identify swallowing sounds. For future research and development, evaluations of patients with dysphagia are necessary to determine the efficacy of the new index for bedside screening of swallowing conditions.

## 1 INTRODUCTION

The increasing population of older patients in recent years has led to an increase in the number of patients with dysphagia; therefore, daily swallowing function assessments along with interventions such as rehabilitation and dietary adjustments are important (Easterling, 2017). Furthermore, these assessments are equally important in patients with diseases, such as amyotrophic lateral sclerosis, that are associated with short‐term dysphagia progression (Dodds et al., 1990).

With improved portability of video endoscopic devices, swallowing evaluations have been increasingly performed outside the hospital setting. However, endoscope insertion during video endoscopy causes pain and discomfort, making it impossible to perform if the patient is uncooperative or unable to endure the procedure. Patients with dysphasia are usually older and often have limited cognitive abilities to communicate effectively during swallowing procedures as seen in patients with dementia and other psychological conditions. This can be a hurdle in administering tests that require patient feedback such as the repetitive saliva swallowing test or the water swallowing test, wherein the patient is instructed to start swallowing and then observed. When cognitive abilities are substantially limited, video endoscopy cannot be performed.

Compared with video fluoroscopy and video endoscopy, cervical auscultation is a simple, minimally invasive method of assessing swallowing function that can be implemented in the patient's usual eating environment. However, as the results of cervical auscultation with an analog stethoscope depend on the practitioner's report, assessments can be subjective and difficult to quantify. Swallowing sounds are generally quite short, lasting approximately 0.7 s from the start of swallowing to the end and are heard as a single block of sound (Dudik et al., 2015; Morinière et al., 2008). Many recent studies have therefore attempted to utilize additional instruments, such as microphones and accelerometers, to evaluate swallowing function and conduct acoustic analyses based on swallowing vibration data (Dudik et al., 2018). Although previous studies measured the number and duration of swallowing sounds, they did not quantitatively assess swallowing strength parameters (Jayatilake et al., 2015; Kamiyanagi et al., 2018). In this study, we combined electronic stethoscope techniques with artificial intelligence (AI) to assemble a system that incorporated sound recognition, with the aim of quantifying swallowing sound.

## 2 METHODS

### 2.1 Participants

Study participants included 20 fifth‐year dentistry student volunteers (10 men and 10 women) who provided written informed consent. All procedures used in the study were approved by the Ethics Committee of Hiroshima University (E‐1599).

### 2.2 Swallowing test protocol

Swallowing sounds were recorded in a silent room. To exclude the effect of noise during the test, participants were given no verbal cues or verbal instructions; instead, using gestures alone, participants were instructed to drink water. At the start of the test, each participant was seated on a reclining dental chair with the back fixed vertically at 90°. They were instructed to drink water in the sitting position. Next, the back of the dental chair was reclined to the horizontal position, and the participant was then instructed to drink the same volume of water in the supine position. The participants drank 10 and 20 ml of water for each posture, with a total of four drinks: 10 and 20 ml in the sitting and supine positions each. The second swallowing sound was analyzed as a comparison. The water was at room temperature and measured with a 20‐ml syringe (ss‐20ESzp, Terumo, Tokyo, Japan), which was injected into the participant's mouth, after which a gesture was made for the participant to start swallowing.

**Figure 1:**
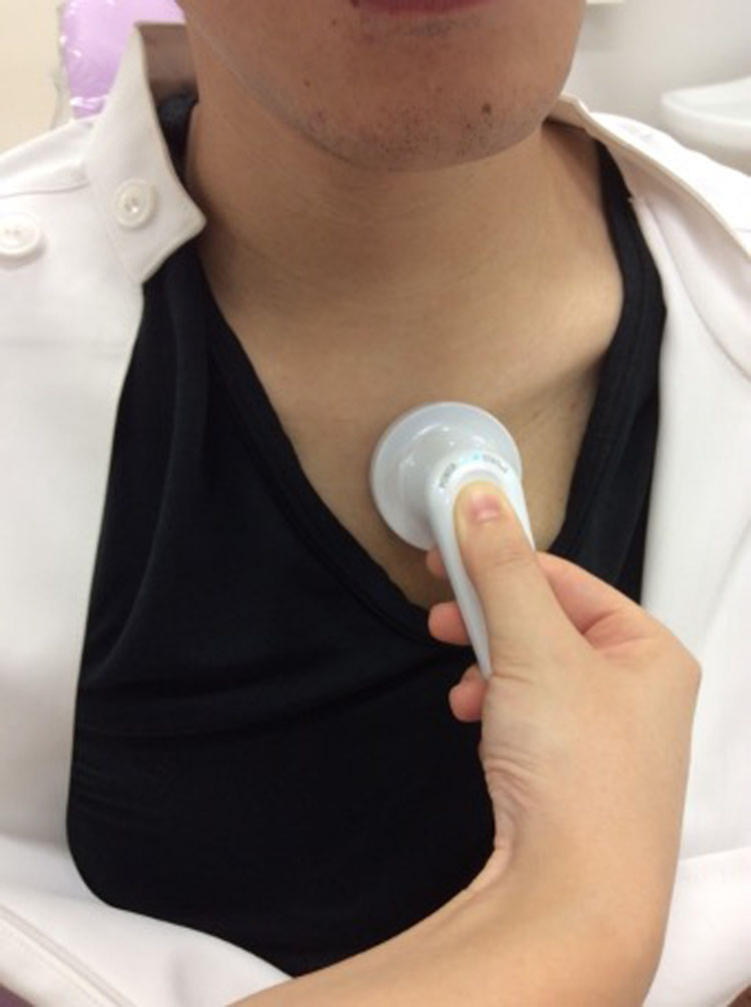
Detection of swallowing sounds from the top of the sternum (beneath the sternal notch) using an electronic stethoscope

**Figure 2:**
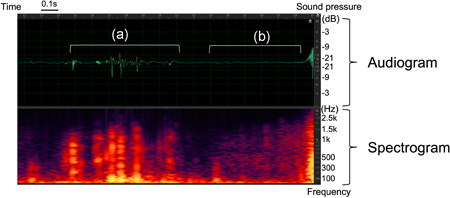
Audiogram and spectrogram of a typical swallowing sound recorded with an electronic stethoscope. Top: Audiogram. Bottom: Spectrogram. (a) Duration of swallowing sound signal. (b) Post‐swallowing expiratory phase

Swallowing sounds were recorded in the 2 Hz to 20 kHz wavelength band using an electronic stethoscope (MSS‐U10C, Pioneer, Tokyo, Japan), which was placed at the top of the sternum beneath the sternal notch (Figure 1). Sound data were transferred in the waveform audio file (WAV) format to a tablet via Bluetooth, and the collected data for the second swallowing cycle (for each volume of water swallowed at each position) were subjected to acoustic analysis at a sampling rate of 8000 Hz using an acoustic analysis software (Audition, Adobe, San Jose, CA, USA). In the acoustic analysis, the acoustic signal was displayed on the computer (Figure 2) and played back to identify the swallowing interval. Swallowing sound pressures [root mean square value (RMS)] were calculated.

### 2.3 Swallowing index calculation using AI

To analyze the sound of bolus inflow, we used an algorithm developed with AI, an adaptive boosting machine learning algorithm that used 50 intermittent sounds (0–4000 Hz) containing a wide frequency band characteristic of the teaching data set because sounds produced during swallowing are the result of anatomical factors and the inflow of the swallowed object, which are heard together as a complex sound. In this case, this complex sound occurs due to the intermittent sounds from water inflow with air. To calculate the sound characteristic (feature value: *y*) of a frame (12 ms), we first derived feature parameters (148 dimensions) for the sound's pitch, intermittency, and continuity using frequency, local variance, and cepstrum analyses. Hyperparameters included frame times and features such as frequency, local variance, and cepstrum analyses; there were 50 training datasets, and the labels were based on the data on which consensus was obtained from three specialists during an auditory evaluation, which were classified into two categories: relevant and not relevant.

Next, machine learning was performed using the teaching data set to calculate feature parameter coefficients. Accordingly, a feature value (*y*) was calculated using the following formula from the feature value parameter of the target auscultation section and the coefficients determined by machine learning.

$$y=\sum_{i=1}^{148}\mathrm{aixi}$$

Feature parameter coefficients calculated by machine learning did not include any bias.

The feature value thresholds (*y*th) were calculated mechanically and determined using receiver operating characteristic (ROC) analysis; the feature values (*y*) calculated for each frame were converted into data based on the presence or absence of target sounds for each frame through comparisons with the feature value thresholds (*y*th) (Zhang & Chen, 2017).

The swallowing index (INDEX) was calculated based on the following formula using the total number of frames in the auscultation section and number of frames assessed as having the target sound. The number of frames increased in direct proportion to duration times.

$$\mathrm{INDEX}\;=\;\frac{\mathrm{No}.\mathrm{of}\;\mathrm{target}\;\mathrm{sound}\;\mathrm{frames}}{\mathrm{Total}\;\mathrm{frames}\;\mathrm{in}\;\mathrm{auscultation}\;\mathrm{section}}\;\times 100$$

In this study, we used this INDEX to perform an acoustic analysis of swallowing sounds.

When evaluating the analysis algorithm, a discriminator that discerned that the target sound existed if the INDEX value was over a certain value was created. Sensitivity, specificity, and accuracy were calculated by comparing the discriminator's assessments with the correct answers. The electronic stethoscope had a contact‐type pressure sensor in the diaphragm to switch on and off at the start and end of the auscultation, respectively. Our system excluded 0.2 s immediately before and after the auscultation from the target auscultation Section.

## 3 RESULTS

The sound of bolus inflow was recorded for 20 volunteers (mean age: 23.5 ± 1.6 years, height: 167.3 ± 8.6 cm, weight: 67.4 ± 17.3 kg). Two participants (both women) were excluded as one had difficulty in drinking in the supine position and we had difficulty recording sounds for the other because of noise caused by her clothing.

**Figure 3:**
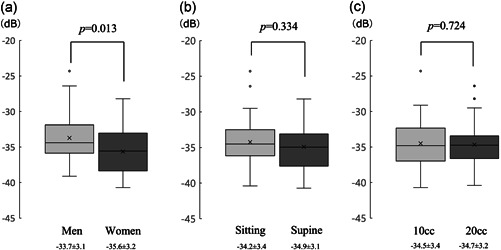
Comparison of changes in sound pressure (root mean square value) of bolus inflow using an acoustic analysis application. (a) Comparison between sexes (men vs. women) using an unpaired *t*‐test. (b) Comparison between postures (sitting vs. supine) using a paired *t*‐test. (c) Comparison between different liquid volumes (10 vs. 20 ml) using a paired *t*‐test. *p*: Hazard ratio

The bolus inflow sound analysis using the acoustic software demonstrated that the RMS value was significantly higher in men than in women (−33.7 ± 3.1 vs. −35.6 ± 3.2 db, *p* < .05). A comparison of the effect of posture on swallowing showed no significant difference in the RMS value (−34.2 ± 3.4 vs. −34.9 ± 3.1 db, *p* = .334). The difference in inflow volume had no significant effect on RMS (−34.5 ± 0.6 [10 ml] vs. −34.7 ± 0.5 db [20 ml], *p* = .72), and there was no detectable change in the bolus inflow sound as a result of the different water volumes (Figure 3).

**Figure 4:**
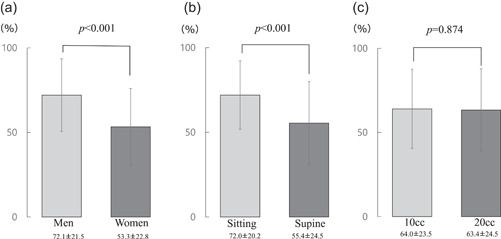
Comparison of changes in the INDEX generated by the artificial intelligence analysis system. (a) Comparison between sexes (men vs. women) using an unpaired *t*‐test. (b) Comparison between postures (sitting vs. supine) using a paired *t*‐test. (c) Comparison between different liquid volumes (10 vs. 20 ml) using a paired *t*‐test. *p*: Hazard ratio

Next, we performed analysis using INDEX with the same swallowing interval as RMS and noted that the INDEX was significantly higher in men than in women (72 ± 21 vs. 53 ± 23, *p* = .001) and significantly lower in the supine position than in the sitting position (72 ± 20 vs. 55 ± 25, *p* < .001). The difference in volume also had no significant effect on the acoustic analysis (64 ± 4 [10 ml] vs. 63 ± 4 [20 ml], *p* = .87) (Figure 4).

## 4 DISCUSSION

Here, we assessed swallowing sounds using the sound recognition function of an AI system, which analyzed bolus inflow sounds recorded with an electronic stethoscope from the top of the sternum. In the conventional cervical auscultation method, the sound is recorded near the cricoid cartilage to obtain the sound of the epiglottis closing and the bolus inflow sound, and bolus passage during swallowing. Because cervical auscultation is easily performed using a stethoscope, it allows for repeated evaluations of swallowing ability in a noninvasive manner. This is convenient for observing a patient's daily eating status and provides important information for screening and dietary adjustment (Watanabe et al., 2020). However, because swallowing sounds are composed of initial clicks and the sound of bolus transfer and because the final pops are generated in <1 s, they can only be heard as a single sound, and consequently, sensory evaluation is difficult (Leslie et al., 2004; Morinière et al., 2008). This study focused on the bolus inflow sound and attempted to quantify swallowing sounds using AI.

Several similar previous studies also evaluated swallowing sounds using a microphone and analyzed their wavelengths, sound pressure, and duration using acoustic analysis software to assess swallowing function; however, research on swallowing sounds has been somewhat unclear; there is a lack of studies on quantitative swallowing strength change assessments due to factors such as sex and posture (Jayatilake et al., 2015; Kamiyanagi et al., 2018). However, we combined the INDEX and swallowing sound pressure (RMS) to compare differences between sexes, postures, and liquid volumes and found that differences in the INDEX were detected between sexes and postures (Figure 4). These results suggest that the combination of an electronic stethoscope and a swallowing assessment algorithm with AI developed by us enabled the bolus inflow sound to be extracted from the complex sounds collected from the top of the sternum.

When assessing swallowing by conventional cervical auscultation, the stethoscope is usually placed close to the cricoid cartilage (Takahashi et al., 1994). However, in this study, we recorded sounds from the top of the sternum beneath the sternal notch (Figure 1). In our preliminary pilot study, we obtained sampling sounds at several different sites, including the lateral side of the cricoid cartilage and above the sternocleidomastoid muscle, but the analysis was affected by noise generated by friction between the skin or clothes and the electronic stethoscope during swallowing movements. Similar noise may have affected analyses in previous studies using laryngeal microphones (Jayatilake et al., 2015; Kamiyanagi et al., 2018). The sternal notch is a good landmark and serves as a stable site for auscultation, and this may be a factor in the relative stability of measurements from recordings made with the electronic stethoscope at the top of the sternum compared with the results of previous studies using recordings made with a laryngeal microphone at the side of the neck. Kuramoto et al. (2020) evaluated swallowing sounds by pattern analysis. We used a machine learning algorithm to evaluate sound quality in short frames and quantify the intensity of swallowing strength (Kuramoto et al., 2020).

Not only swallowing sounds but also physiological sounds are propagated through three routes: soft tissue, hard tissue (bone), and air. If auscultation is performed above the soft tissues of the neck, swallowing sounds are obtained via soft tissue propagation (Andrès et al., 2018). The top of the sternum, the site used for auscultation in this study, is anatomically close to the main bronchi, meaning that bone propagation through the tracheal cartilage and air propagation through the bronchial lumen are both present; in anatomical terms, this makes it a better site for swallowing sound propagation than the side of the neck. Respiratory sounds were also recorded in the WAV format immediately following swallowing sound assessments (Figure 2). These sounds together indicated that swallowing and respiratory sounds can be analyzed simultaneously, enabling the assessment of swallowing and respiration coordination. For some experiments, researchers place the transducers at the suprasternal notch. The electronic stethoscope used in this study is fitted with a sensing function for respiratory sounds, and we intend to develop swallowing analysis algorithms for conditions such as deglutition apnea in the future.

This study has several limitations. First, it included only healthy adult volunteers. Previous studies have found that bolus inflow sound in patients with dysphagia exhibits different characteristics depending on the pathological conditions and these reportedly differ from those of healthy adults. Further studies that assess bolus inflow sounds from patients with dysphagia are required (Dudik et al., 2018). Second, the only test liquid used in this study was water. Therefore, the results may not be applicable to other liquids containing different properties and characteristics when conducting swallowing index analyses using the AI algorithm developed in this study. In such cases, a different analysis algorithm must be used for each substance ingested. Third, this study did not include comparisons with findings of other investigations such as those that utilized video fluoroscopy and video endoscopy. Future comparative studies that conduct detailed comparisons with other swallowing functional assessment methods are required, particularly for patients with dysphagia. Fourth, there is a risk of overfitting; the data set is divided into training data set and test data set: the former is used for development and the latter for performance evaluation. As a result, a positive diagnosis rate of ≥0.8 was achieved, and we believe that there is no significant performance degradation due to overfitting at this point. Cross‐validation is a topic that needs to be further discussed in the future.

## 5 CONCLUSIONS

We demonstrated the value and efficacy of the combined use of an electronic stethoscope and AI for the objective quantification of swallowing sound. Future studies are necessary for confirming the clinical efficacy of this system to complement the bedside screening of swallowing strength.

## CONFLICT OF INTERESTS

The authors declare that there are no conflict of interests.

## AUTHOR CONTRIBUTIONS

All authors contributed to the study conception and design. Research design and investigation was performed by Yoshitaka Shimizu, Noboru Saeki and Nobuaki Shime, Data acquisition and analysis was performed by Kazuma Suzuki, Kana Oue and Takuma Sadamori. Interpretation and preparation of the manuscript was performed by Yasuo Tsutsumi, Shinichiro Ohshimo, and Masahiro Irifune. All the authors read and approved the final manuscript.

6

## Data Availability

The data that support the findings of this study are available from the corresponding author upon reasonable request.
